# On the reduction of aperture complexity in kidney SABR

**DOI:** 10.1002/acm2.13215

**Published:** 2021-03-23

**Authors:** Mathieu Gaudreault, Keith Offer, Tomas Kron, Shankar Siva, Nicholas Hardcastle

**Affiliations:** ^1^ Department of Physical Sciences Peter MacCallum Cancer Centre Melbourne Vic. Australia; ^2^ Sir Peter MacCallum Department of Oncology The University of Melbourne Melbourne Vic. Australia; ^3^ Department of Radiation Oncology Peter MacCallum Cancer Centre Melbourne Vic. Australia; ^4^ Centre for Medical Radiation Physics University of Wollongong Wollongong NSW Australia

**Keywords:** FFF, interplay, kidney, SABR, VMAT

## Abstract

**Background:**

Stereotactic ablative body radiotherapy (SABR) of primary kidney cancers is confounded by motion. There is a risk of interplay effect if the dose is delivered using volumetric modulated arc therapy (VMAT) and flattening filter‐free (FFF) dose rates due to target and linac motion. This study aims to provide an efficient way to generate plans with minimal aperture complexity.

**Methods:**

In this retrospective study, 62 patients who received kidney SABR were reviewed. For each patient, two plans were created using internal target volume based motion management, on the average intensity projection of a four‐dimensional CT. In the first plan, optimization was performed using a knowledge‐based planning model based on delivered clinical plans in our institution. In the second plan, the optimization was repeated, with a maximum monitor unit (MU) objective applied in the optimization. Dose‐volume, conformity, and complexity metric (with the field edge metric and the modulation complexity score) were compared between the two plans. Results are shown in terms of median (first quartile — third quartile).

**Results:**

Similar dosimetry was obtained with and without the utilization of an objective on the MU. However, complexity was reduced by using the objective on the MUs (modulation complexity score = 0.55 (0.50–0.61) / 0.33 (0.29–0.36), *P*‐value < 10^−10^, with/without the MU objective). Reduction of complexity was driven by a larger aperture area (area aperture variability = 0.68 (0.64–0.73) / 0.42 (0.37–0.45), *P*‐value < 10^−10^, with/without the MU objective). Using the objective on the MUs resulted in a more spherical dose distribution (sphericity 50% isodose = 0.73 (0.69–0.75) / 0.64 (0.60–0.68), *P*‐value < 10^−8^, with/without the MU objective) reducing dose to organs at risk given respiratory motion.

**Conclusions:**

Aperture complexity is reduced in kidney SABR by using an objective on the MU delivery with VMAT and FFF dose rate.

## 1 INTRODUCTION

Stereotactic ablative body radiotherapy (SABR) is a novel treatment to treat patients with renal cell carcinoma (RCC) for whom surgery is not an option. It results in excellent local control and low toxicity rates in primary RCC.^1^, ^2^ This technique is noninvasive and delivered in an outpatient procedure. Moreover, kidney SABR treatment is not limited to tumor size or kidney position as is the case with other alternatives to surgery such as radiofrequency ablation or cryoablation.^3^

As the tumor moves during treatment with respiratory motion, the interplay between the moving multileaf collimator (MLC) and tumor motion may result in discrepancies between planned and delivered doses. Several studies have demonstrated limited impact of the interplay effect on conventionally fractionated treatments, as any discrepancies are averaged out during the course of treatment.^4^, ^5^, ^6^, ^7^, ^8^, ^9^, ^10^ As opposed to conventionally fractionated treatments, interplay effect may impact SABR treatments because the number of fractions is typically 1–5. ^10^, ^11^ Strategies to minimize interplay effect include reducing the aperture complexity, ^11^, ^12^ increasing the number of beams and fractions, ^10^, ^12^ reducing dose rate, ^12^, ^13^ decreasing tumor amplitude ^13^, ^14^ and breathing cycle ^10^, ^11^, ^12^, ^15^ and treating with higher dose per fraction. ^10^, ^12^, ^16^

Contemporary kidney SABR treatments are often treated with volumetric modulated arc therapy (VMAT), often due to the challenging geometric relationship between the target and adjacent organs at risk (OAR). Furthermore, flattening filter‐free (FFF) delivery is highly attractive to reduce the treatment time via increased dose rates. Due to substantial reductions in beam‐on time with high dose rates, and the use of ultra‐hypofractionated regimens, the interplay effect may impact the fidelity of the planned treatment dose. Furthermore, reduction of aperture complexity and reduction of required monitor units (MUs) are of interest to reduce the treatment delivery time, which may be particularly important in the context of respiratory gating or breath hold treatment delivery.

We have recently shown that reduction in aperture complexity can be achieved in lung SABR by using an optimization objective on the total number of MUs, referred as the “MU objective” in the Eclipse treatment planning system.^17^ The MU objective has been available in the Eclipse treatment planning system for RapidArc optimization since version 8.5. Previous work established use of the MU objective results in reduction in the total MUs, and therefore in the beam‐on time, while preserving adequate dosimetry by using the MU objective in prostate,^18^, ^19^ head and neck,^18^, ^20^ gynecological,^18^ and lung SABR.^21^ However, the impact on the aperture complexity by using the MU objective was not addressed in these previous studies.

The purpose of this study is to determine the dosimetric impact of reducing aperture complexity via inclusion of a penalty on total MUs in the optimization on kidney SABR VMAT treatment plans. It is hypothesized that substantial reductions in aperture complexity can be achieved with minimal impact on dosimetric quality in kidney SABR.

## 2 MATERIALS AND METHODS

We included consecutive 62 patients with primary RCC treated with SABR between 2012 and 2018 at our institution. Fractionation was 26 Gy in a single fraction for lesion size smaller or equal to 4 cm and 42 Gy in three fractions for lesion size larger than 4 cm.^22^ Out of the 62 patients, 23 patients received 26 Gy in a single fraction and 38 patients had 42 Gy in three fractions. One patient was treated at 18 Gy in one fraction, but was replanned in this study with 26 Gy in one fraction. These patients were treated with 3D conformal radiation therapy, intensity‐modulated radiation therapy or VMAT. Ethics approval for this study was provided by Peter MacCallum Cancer Centre.

Each patient was simulated using a four‐dimensional CT scan (4DCT). The tumor was segmented on all respiratory phases. Gross tumor volume (GTV) was accumulated on the average intensity projection (AIP) of the 4DCT to generate an internal target volume (ITV). A planning target volume (PTV) was created using an isotropic 5 mm expansion of the ITV. The AIP of the 4DCT was used for contouring, planning, optimizing, and calculating the dose distribution. OARs were segmented on the AIP depending on the extent of respiratory motion. OAR contours in some cases overlap with the ITV contour in cases where the OAR is proximal to the tumor.

All patients were replanned for the purpose of this study. Plans were generated by using the Eclipse treatment planning system (Varian Medical Systems, Palo Alto, CA, USA) with AcurosXB Algorithm (v15.6.06) reporting dose to medium for dose calculation and Photon Optimization Algorithm (v15.6.06) for optimization.

Two coplanar ipsilateral arcs of 210^o^ were used for each plan with the arc rotating from the posterior–anterior direction around to 30° past midline. The isocenter was placed at the centroid of the PTV. Collimator angles were set at 5°/355°. A clinical 10 MV‐FFF beam model with maximum dose rate 2400 MU/min was used with the HD 120 MLC.

Dose objectives to target and constraints to normal tissue used for optimization were applied by using a knowledge‐based planning (KBP) model (RapidPlan v15.5.11 Varian Medical Systems, Palo Alto, CA, USA). The model was constructed from 53 clinical kidney SABR plans delivered at our institution. An upper point dose objective was added to certain OARs to control the high dose region. Parameters used in the model are shown in Table 1. The normal tissue objective (NTO) was set to “Automatic NTO” with a priority of 150.

**Table 1:** Knowledge‐based planning model optimization objectives.

Structure	Objective	Volume (%)	Dose (%)	Priority
ITV	Upper	0	130	100
ITV	Lower	2	125	100
ITV	Lower	100	110	100
PTV‐ITV	Upper	0	110	100
PTV‐ITV	Lower	100	100	120
Kidney_I	Line			
Kidney_C	Line			
SmallBowel_prox	Line			
SmallBowel_prox	Upper	0	70	100
LargeBowel	Line			
LargeBowel	Upper	0	100	100
Skin	Line			
Skin	Upper	0	70	100
Liver	Line			
Stomach	Line			
Stomach	Upper	0	70	100
SpCord	Line			
SpCord	Upper	0	40	100

The primary method of reducing aperture complexity was an objective on the upper value of MU. Once selected, this parameter defines a range of targeted MU for the plan. The optimizer penalizes any MU outside the desired interval. The penalty is weighted by a strength assigned to the MU objective. This parameter takes value between 0 and 100. In addition to the MU objective, the aperture shape controller was set to “Very High”, the convergence mode to “On", and the multiresolution (MR) level at restart to “MR3” for all plans.

Optimization and calculation was a two‐step process to determine the upper value of the MU objective. The original MU was first obtained by optimizing and calculating the dose without the MU objective in one single process without user interaction. The plan was then normalized so that 100% of the prescription dose covers 95% of the target volume and is referred as “NMUO” plan in this study.

The NMUO plan was copied and an objective on the MU was added. The upper value of the MU objective was set to 50% of the original total MUs with a strength of 70, and the plan was then optimized from scratch through one process without user interaction. The plan was also normalized so that 95% of the target volume is covered with 100% of the prescription, referred as “MUO” plan.

Any plan for which a dose constraint was not respected was replanned by adjusting the objectives to respect dose limits. These replans are referred as “modified KBP” as opposed with “original KBP” plan. These were all for cases in which an OAR was close to or overlapping with the target, and were identified as those with challenging plan geometry. If a structure was overlapping with the PTV or the ITV, the PTV and ITV structures were cropped to generate an optimization structure. In two patients, the two partial arcs of 210^o^ had to be modified to two full rotation arcs of 358°. The two calculation steps method was repeated by modifying the upper dose limit to the organ up to the point where all dose limits were respected. Where possible while meeting OAR constraints, the plans were normalized to 95% of the target volume was covered by the prescription dose. Where this was not possible, loss to target coverage was accepted to ensure OAR constraints were respected.

Dose metrics for the target and OARs, shown in Table 2, were evaluated. Dose limits were based on QUANTEC recommendations.^23^, ^24^, ^25^ Plan generation, optimization, calculation, and metrics extraction were done by using the Eclipse Scripting Application Programming Interface (ESAPI). Plan conformity was determined using the RTOG conformity index, defined as the reference isodose volume divided by the target volume.^26^ The 95% isodose was used as reference isodose to calculate the conformity index (CI95). A value of CI95 = 1 indicates ideal conformation. The target was partially irradiated if CI95 < 1 while the irradiated volume was greater than the target volume if CI95 > 1.^27^ Moreover, acceptable CI95 values were defined as values smaller than 1.2 while minor deviations were defined for values greater than 1.2 but smaller than 1.5.^28^ Conformity of the low dose region was assessed with the CI50, or equivalently the R50, defined as the 50% isodose volume divided by the target volume. CI50 conformity deviation was assessed through the ALARA principle with a planning goal of CI50 < 5 for all PTV volumes.

**Table 2:** Normal dose tissues constraints used.

Organ	26 Gy/1Fx	42 Gy/3Fx
Spinal cord	D0.03cc < 12 Gy	D0.03cc < 18 Gy
Skin	D1.5cc < 18 Gy	D1.5cc < 24 Gy
Small bowel	D30cc < 12.5 Gy	D0.03cc < 30 Gy
Large bowel	D1.5cc < 26 Gy	D1.5cc < 42 Gy
Stomach		D0.03cc < 30 Gy
	D5cc < 22.5 Gy	D5cc < 22.5 Gy
liver	No constraint	D700cc < 15 Gy
Contralateral kidney	V10 Gy < 33%	V10 Gy < 33%

Due to the proximity of bowel structures to the target, and their variation in position between treatment planning and each treatment session, it is desirable to minimize higher isodose lines extending between bowel loops which may arise as a consequence of using the bowel structures for optimization. This was assessed by calculating the sphericity of the isodoses lines. The 100% and the 50% isodose lines were converted to contours and exported. Pyradiomics v3.0^29^ was used to calculate the sphericity of the contour. The resulting value ranged between 0 and 1, where 1 indicated a perfect sphere.

Robustness of the plans were measured by calculating the edge metric (EM) and the modulation complexity score (MCS). EM was calculated according with C_1_ = 0 and C_2_ = 1.^30^, ^31^, ^32^ In this representation, EM reports the y‐leaf sides normalized by the area aperture weighted per control point. Plan complexity decreases as EM decreases to 0. MCS was interpreted according to McNiven et al.^33^ and used by others.^31^, ^34^ MCS is a score based on adjacent leaf sides, measured by leaf segment variability (LSV), and on the area of the aperture, measured by the area aperture variability (AAV). For a given control point, the LSV is proportional to the average position difference between adjacent leaves over all leaves contributing to the open field normalized by the difference between maximal position and the minimal position overall leaves within a bank. The AAV is the aperture area normalized by the maximum aperture area over all control points. LSV and AAV were also weighted per control point and defined as LSVw and AAVw. The three quantities take value between 0 and 1. Plan complexity decreases as MCS, LSVw, and AAVw increases to 1. The weights per control point used were the same for calculation of EM and MCS.^30^ Both plan complexity metrics were calculated from an in‐house script. Details of the calculation are shown in the appendices. To provide a lower bound of complexity reference point, the beams in the MUO plan were converted to dynamic conformal arc therapy (DCAT) fields by fitting the aperture to the PTV in each control point. The complexity metrics EM and MCS, including LSVw and AAVw, were computed for the DCAT plans.

The average difference of each metrics between plan with and without the MU objective was calculated and a Wilcoxon signed‐rank test was performed to determine the statistical significance of the median difference by using Scipy v1.5.2. The null hypothesis was rejected if the *P*‐value was less than 0.05 (5% significance level). Statistical quantities are reported in terms of median (first quartile – third quartile).

## 3 RESULTS

### 3.1 Dosimetry

**Fig. 1:**
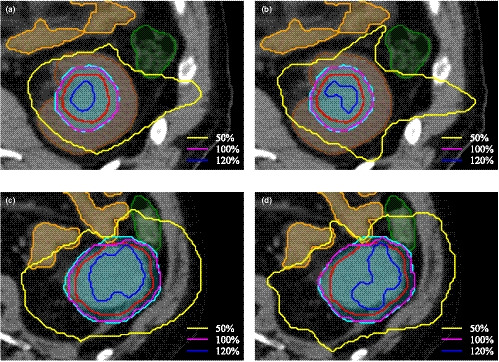
Axial view of a typical dosimetry obtained for one patient with original KBP (top row) and one patient with modified KBP (bottom row). Isodoses 50% (yellow), 100% (magenta), and 120% (blue) are shown for plan with the MU objective (a) and (c) and without the MU objective (b) and (d). The structures ITV (red), PTV (cyan), nontumor ipsilateral kidney (brown), small bowel within 5 cm of the ITV (orange), and large bowel (green) are shown.

An example of a typical dose distribution in the axial plane is shown in Fig. 1 for an original KBP plan (top row) and a modified KBP plan (bottom row) obtained with the MU objective (a) and (c) and without the MU objective (b) and (d). PTV volumes for the whole cohort ranged from 25 cc to 390 cc with a median of 131 cc.

**Fig. 2:**
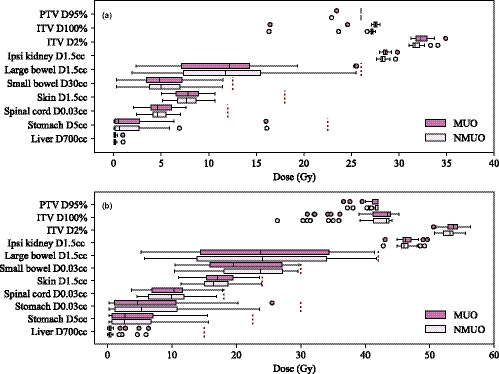
Comparison of dose metrics with (MUO) and without (NMUO) the MU objective for patients with fractionation (a) 26 Gy/1 Fx (24 patients) and (b) 42 Gy/3 Fx (38 patients). Red dotted lines indicate dose limit.

Resulting dose metrics for the single fraction cohort are shown in Fig. 2(a). ITV coverage by the 100% isodose and the ITV hotspot were slightly higher in plans with the MU objective (ITV D100% = 27.5 (27.4–27.7) Gy / 27.1 (27.0–27.3) Gy, *P*‐value < 10^−5^ and ITV 2% = 32.2 (31.8–32.9) Gy / 31.7 (31.5–32.2) Gy, *P*‐value < 10^−5^, MUO/NMUO).

The addition of the MU objective did not significantly change most OAR dose metrics. Near maximum dose to the ipsilateral kidney was slightly higher when the MU objective was used (ipsilateral kidney D1.5cc = 28.6 (28.3–28.8) Gy / 28.2 (28.1–28.5) Gy, *P*‐value < 10^−6^, MUO/NMUO). There were no statistically significant differences in dose metrics for the remaining OARs (*P*‐value = [0.2,0.65]).

In one patient the dose limit to the large bowel was not respected due to overlap with the ITV. Coverage to the PTV and ITV was slightly higher with the MU objective for this modified KBP plan (PTV D95% = 23.4 Gy / 22.9 Gy, MUO/NMUO and ITV D100% = 16.4 Gy / 16.3 Gy, MUO/NMUO). For this patient, the dose to ipsilateral kidney, large bowel, and skin increased with MUO while dose metrics for the small bowel, stomach, and liver were decreased with MUO.

Dose metrics for the whole multifraction cohort are shown in Fig. 2(b). Coverage to the PTV and ITV was similar irrespective of use of the MU objective (PTV D95% = 42.0 (41.1–42.0) Gy / 42.0 (41.6–42.0) Gy, *P*‐value < 10^−2^ and ITV D100% = 43.4 (41.2–43.9) Gy / 43.3 (41.2–43.7) Gy, *P*‐value < 10^−2^, MUO/NMUO). Near maximum dose to the ITV was also similar with and without the MU objective (ITV D2% = 53.7 (52.9‐54.3) / 53.1 (52.1‐53.6) Gy, *P*‐value < 10^−3^, MUO/NMUO).

Interestingly, near maximum dose to the small bowel was reduced in some cases by using the MU objective but the differences were not statistically significant (small bowel D0.03cc = 19.2 (15.4‐27.1) Gy / 23.7 (18.1‐27.2) Gy, *P*‐value = 0.08, MUO/NMUO). The near maximum spinal cord dose increased with application of the MU objective but remained below the dose limit (spinal cord D0.03cc = 10.3 (6.9–11.6) Gy / 9.9 (6.4–11.8) Gy, *P*‐value = 0.01, MUO/NMUO). Near maximum dose to the ipsilateral kidney was similar between the two plans (ipsilateral kidney D1.5cc = 46.2 (45.9–47.1) Gy / 46.0 (45.6–46.7) Gy, *P*‐value < 10^−2^, MUO/NMUO). Application of the MU objective did not make a statistical significant difference for other OARs (*P*‐value = [0.17,0.75]).

KBP plans required modification for 15/38 patients in the multifraction group to meet dose constraints. Refinement of the objectives was required to meet spinal cord constraint in one patient. Dose limit to the skin was not respected in two patients. These were two left‐sided tumor exophytic close to the patient skin. Small bowel was overlapping with the target in seven patients, large bowel was overlapping the ITV in three patients, and both the small and the large bowel were overlapping the ITV in two patients.

Optimization to make modified KBP plans clinically deliverable resulted in a loss of coverage to the target (PTV D95% = 40.9 (40.0‐41.6) Gy / 41.4 (40.7‐41.8) Gy, MUO/NMUO, *P*‐value < 10^−2^) as opposed with original KBP plans (PTV D95% = 42 Gy for all plans MUO/NMUO). Use of the MU objective increased the ITV D100% and ITV D2% in the original KBP group (ITV D100% = 43.8 (43.6‐44.1) Gy / 43.5 (43.3–43.7) Gy, *P*‐value < 10^−2^ and ITV 2% = 53.0 (52.6–53.7) Gy / 52.4 (51.7–53.3) Gy, *P*‐value < 10^−2^, MUO/NMUO). Differences in the ITV D100% and ITV D2% medians were not significant in the modified KBP group (*P*‐value = 0.18 and 0.06, respectively).

Near maximum dose to the small bowel was close to the dose limit in modified KBP plans with 60% (9/15) of plans having coverage reduced to meet this limit (small bowel D0.03cc = 28.8 (23.8–29.6) Gy / 28.5 (25.4–28.9) Gy, *P*‐value = 0.42, MUO/NMUO). However, the near maximum small bowel dose was reduced for original KBP plan with the MU objective (small bowel D0.03cc = 17.4 (13.5–19.2) Gy / 21.7 (17.0–23.9) Gy, *P*‐value = 0.01, MUO/NMUO).

The only significant statistical difference in dose metric medians of modified KBP plans was for PTV D95% (other *P*‐value = [0.06,0.93]). Dose metric median values were systematically higher in all OARs except small bowel in original KBP plans with the MU objective, but still far from any dose limit. Near maximum spinal cord dose was larger with the MU objective (spinal cord D0.03cc = 8.6 (6.5–11.6) Gy / 7.1 (5.9–11.3) Gy, *P*‐value = 0.01, MUO/NMUO) while differences to the stomach was similar with and without the MU objective (stomach D5cc = 2.62 (0.4–6.9) Gy / 2.60 (0.4–6.8) Gy, *P*‐value = 0.04, MUO/NMUO). Other differences in original KPB were not statistically significant (*P*‐value = [0.09,0.72]).

### 3.2 Conformity Index and sphericity

**Fig. 3:**
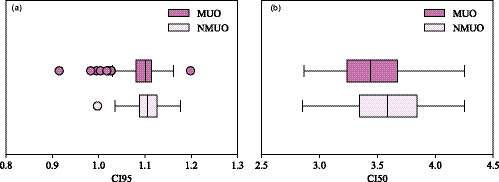
Conformity index (a) CI95 and (b) CI50 shown with (MUO) and without (NMUO) the MU objective.

CI95 is shown in Fig. 3(a). The CI95 median and interquartile range were similar with and without the MU objective (CI95 = 1.10 (1.08–1.11) / 1.11 (1.09–1.13), *P*‐value < 10^−5^, MUO/NMUO). CI95 was smaller in modified KBP plans when using the MU objective (CI95 = 1.03 (1.01‐1.08) / 1.08 (1.06‐1.10), *P*‐value < 10^−3^, MUO/NMUO) while it was similar in original KBP plans (CI95 = 1.11 (1.09–1.12) / 1.11 (1.09–1.13), *P*‐value < 10^−2^, MUO/NMUO). One plan had CI95 < 1 without the MU objective and three plans had CI95 < 1 with the MU objective, all for modified KBP plans in the multifraction cohort. All values of CI95 satisfied the planning objective (CI95 < 1.2) with and without the MU objective.

CI50 was reduced when using the MU objective (CI50 = 3.4 (3.2–3.7) / 3.6 (3.3–3.8), *P*‐value < 10^−7^, MUO/NMUO), as shown in Fig. 3(b). The same conclusion was obtained with modified KBP plans (CI50 = 3.3 (3.1–3.5) / 3.5 (3.3–3.7), *P*‐value < 10^−2^, MUO/NMUO) and original KBP plans (CI50 = 3.5 (3.3–3.7) / 3.6 (3.4–3.9), *P*‐value < 10^−5^, MUO/NMUO). All values of CI50 were smaller than the planning objective (CI50 < 5) with and without the MU objective.

**Fig. 4:**
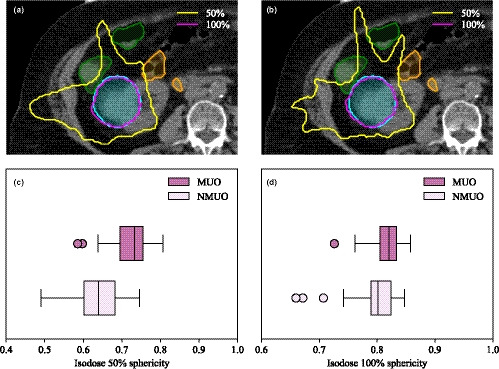
Isodoses 50% (yellow) and 100% (magenta) for a plan (a) with and (b) without the MU objective. The large bowel (green) and small bowel (orange) are adjacent to the PTV (cyan) resulting in the 50% isodose line splaying in between bowel loops. Dose splay is reduced by using an objective on the monitor unit. Sphericity of the (c) 50% isodose and (d) 100% isodose.

Isodoses were more spherical when using the MU objective. An example of the 100% and 50% isodoses for the patient with the worst isodose 50% sphericity in NMUO plan is shown in Fig. 4(a) with and in Fig. 4(b) without the MU objective. A significant increase in the isodose 50% sphericity was observed when using the MU objective (sphericity 50% isodose = 0.73 (0.69–0.75) / 0.64 (0.60–0.68), *P*‐value < 10^−8^, MUO/NMUO) as shown in Fig. 4(c). This effect was also observed with the 100% isodose, as shown in Fig. 4(d), but to a lower extent (sphericity of 100% isodose = 0.82 (0.80–0.83) / 0.80 (0.79–0.82), *P*‐value < 10^−8^, MUO/NMUO). The same conclusions hold for both modified and original KBP plans.

### 3.3 Aperture complexity

Using the objective on the MU yielded to a reduction in the total MU for both the single fraction group (total MU = 5617 (5164–5910) / 10439 (9560–11237), *P*‐value < 10^−6^, MUO/NMUO) and the multifraction group (total MU = 3022 (2619‐3238) / 5818 (5064‐6428), *P*‐value < 10^−7^, MUO/NMUO).

**Fig. 5:**
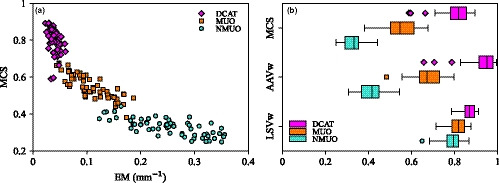
(a) Modulation complexity score (MCS) vs edge metric (EM) in mm^−1^ with (MUO) and without (NMUO) the MU objective. Complexity of DCAT fields is shown for comparison. (b) Modulation complexity score (MCS), area aperture variability weighted by monitor unit (AAVw), and leaf sequence variability weighted monitor unit (LSVw) with and without the MU objective and for DCAT fields.

Using the objective on the total MUs also reduced the aperture complexity, as measured by EM and MCS shown in Fig. 5(a). Results are compared with the aperture complexity of their DCAT counterpart in the figure. Median and interquartile range of the distribution and statistical significance are reported in Table 3. Multifractionation subset plans were less complex than the single fraction subset plans. The type of fractionation was based on lesion size with the PTV larger in the multifractionation subset. This leads to a larger aperture area to cover the target and to a smaller EM and larger MCS. Using the MU objective decreased aperture complexity (relative difference of the medians with respect to NMUO plan was −61% and 64% in EM and MCS, respectively, both *P*‐value < 10^−8^). The two regimes are shown in Fig. 5(b). Even if some modulation is reduced in MUO plan, they are still more complex than their DCAT counterpart (relative difference of the medians in complexity of CF with respect to MUO plan was −61% and 50% in EM and MCS, respectively, both *P*‐value < 10^−10^).

**Table 3:** Edge metric (EM), modulation complexity score (MCS), aperture area variability weighted (AAVw), and leaf sequence variability weighted (LSVw) for MUO and NMUO plan and for the single‐ and multifractionation group. Results are reported in terms of median (first quartile – third quartile).

		n	EM	MCS	AAVw	LSVw
Cohort	MUO	62	0.10 (0.07‐0.14)	0.55 (0.50‐0.61)	0.68 (0.64‐0.73)	0.82 (0.79‐0.85)
	NMUO	62	0.25 (0.20‐0.30)	0.33 (0.29‐0.36)	0.42 (0.37‐0.45)	0.80 (0.76‐0.82)
	*P*‐value		<10^−10^	<10^−10^	<10^−10^	<10^−10^
1 Fx	MUO	24	0.13 (0.10‐0.15)	0.52 (0.50‐0.59)	0.68 (0.65‐0.72)	0.79 (0.76‐0.81)
	NMUO	24	0.29 (0.26‐0.33)	0.32 (0.29‐0.35)	0.43 (0.37‐0.45)	0.76 (0.73‐0.78)
	*P*‐value		<10^−6^	<10^−6^	<10^−6^	<10^−6^
3 Fx	MUO	38	008 (0.07‐0.10)	0.58 (0.52‐0.62)	0.68 (0.62‐0.73)	0.84 (0.82‐0.86)
	NMUO	38	0.22 (0.19‐0.26)	0.34 (0.30‐0.37)	0.42 (0.37‐0.45)	0.81 (0.80‐0.83)
	*P*‐value		<10^−7^	<10^−7^	<10^−7^	<10^−7^

The impact of using an objective to the MU is explained by AAVw and LSVw. These two quantities are shown in Fig. 5(b) while the median and interquartile range of the distribution and statistical significance are detailed in Table 3. Reduction in complexity was driven by a larger area aperture with the MU objective while leaf traveling changed minimally. This was expected as the parameter controlling the leaf travel, the so‐called aperture shape control, was the same in both subsets. Using an objective on the MU reduces the degree of freedom available to the optimizer as the MUs were constrained. This loss was compensated by a larger aperture area. Therefore complexity was reduced.

## 4 DISCUSSION

Results suggest that aperture complexity is minimized in kidney SABR treatment by using VMAT with FFF dose rate and an objective on the MU. Using an objective on the upper value MU forced the optimizer into using a larger aperture area. Since leaf travel was kept constant, aperture area increase resulted to a reduction in plan complexity. Importantly, there was minimal dosimetric compromise when using the MU objective. Using the objective on the MU was even beneficial to the small bowel in the multifraction group for original KPB plan. Furthermore, constraining the MUs resulted in more spherical isodoses which reduced the conformity indices and dose splay between bowel loops was reduced. This is particularly important with respect to positional variation of small and large bowel loops at time of treatment.

The main advantage of the objective on the MU is the beam‐on time reduction that follows from the MU reduction (50% reduction in this study). Value of the upper limit on the MU objective and its associated strength were not optimized in this study, but were derived from previously investigated values in lung SABR.^35^ The level of complexity achieved with VMAT and DCAT fields were similar in this previous study. This was not the case in this work as VMAT plans with the MU objective in use were still more complex than DCAT fields. This difference between the two studies may be due to the parameters used in the application of the MU objective (Max MU = 50% and strength = 70 in this study as opposed with Max MU = 40% and strength = 80 in the previous study). Furthermore, the planning process used in this work required optimization of a plan without the MU objective, and subsequent reoptimization using the original MUs to derive the MU objective setting. A method involving a fixed upper limit to the MU objective per site and fraction would be of interest. Our results showing the MU distribution in MUO plans may be useful to start optimization directly with the second step in the optimization used in this work.

Modification to the KBP model had to be performed in 26% (16/62) of the patients. PTV and ITV were cropped and the resulting structures used as optimization structures in 13 patients. As a result, target coverage was reduced with the use of the MU objective in the multifraction group. OAR dosimetry was similar with and without the MU objective and all differences in dose metric medians were not statistically significant. These plans involved complex geometry but the conformity indices were lowered, isodoses were more spherical and the aperture complexity was reduced when using the MU objective. Allowing increased complexity for these plans might be needed to achieve the same plan quality as original KBP plan. Utilization of an objective on the MU is recommended for plans with less complex target–OAR relationship, however, where there is more complex geometry, increased modulation may be required to meet plan objectives.

There are some limitations in this study. The shape of the GTV was preserved on each phase although the GTV might deform with motion. Modification to the GTV contour might affect the ITV volume resulting from the accumulation method. Consequently, the ITV may overlap with a surrounding OAR which would require modification to the KBP model. Furthermore, OAR contours used in this study were the clinical contours on the AIP image of the 4DCT. All OARs could have been delineated on each respiratory phase and accumulated on the AIP image. This process would have resulted in more patients with overlapping ITV and OAR and hence requiring manual modification of the KBP plan to meet objectives.

## 5 CONCLUSION

In conclusion, kidney SABR plan quality is optimal by using an objective on the upper value of the MU with VMAT and FFF dose rate. This combination leads to a similar dosimetry compared with plan without the MU objective. Isodoses are more spherical with the MU objective which reduces dose splay between OAR. Moreover, the open aperture area is larger when using the MU objective which reduces the aperture complexity. Finally, the MUs are significantly reduced with the objective on the MU, which reduces the treatment time and the probability of intrafraction variation between plan and delivery.

## CONFLICT OF INTEREST

This research was partially funded by Varian Medical Systems.

## Author Contribution

Mathieu Gaudreault: Conceived and designed the analysis, collected the data, performed the analysis, and wrote the paper. Keith Offer: Contributed to analysis tool. Tomas Kron: Conceived and designed the analysis. Shankar Siva: Conceived and designed the analysis. Nicholas Hardcastle: Conceived and designed the analysis and wrote the paper. All authors discussed the results and commented the manuscript.

## Appendix A Edge metric calculation

Edge metric for a beam EM_beam_ was calculated according to numerous literature works,^30^, ^31^, ^32^

(A1) $${\text{EM}}_{\text{beam}}=\sum_{\text{cp}=1}^{N}W\left[\text{cp}\right]\left(\frac{C_{1}x\left[\text{cp}\right]+C_{2}y\left[\text{cp}\right]}{A\left[\text{cp}\right]}\right),$$

where the sum runs over all the control points cp within the beam, C_1_ and C_2_ are weighting constants, x[cp] is the x‐leaf end, y[cp] is y‐leaf side, A[cp] is the aperture area and W[cp] is the aperture weight. EM for the plan was obtained by weighting EM_beam_ with respect to the monitor unit MU of this beam, namely

(A2) $$\text{EM =}\;\sum_{\text{beam}}{\text{EM}}_{\text{beam}}\frac{{\text{MU}}_{\text{beam}}}{{\text{MU}}_{\text{total}}}\text{.}$$

The same representation of Eq. (A1) as recommended in the literature^30^, ^31^, ^32^ was adopted, namely C_1_ = 0 and C_2_ = 1. In this representation, only the y‐leaf side contributes to Eq. (A1). Leaves in the field were defined as all leaves not under a jaw. A leaf was considered in the field if it was partially covered by a jaw. For each control point, the area of leaf pair was calculated such as the distance between the two leaves constituting a pair multiplied by their widths (w). Only the fraction of the leaf width (f) inside the field was considered if a leaf pair was partially covered by a jaw. All leaf pairs not overlapping with the jaw had f = 1. EM was expressed in mm^−1^.

## Appendix B MCS calculation

Modulation complexity score was calculated along the lines of previous literature works.^31^, ^33^, ^34^ For each control point, open leaves not under a jaw were considered inside the field, with total number N_F_. A pair of leaf was considered open if the distance between two opposite leaves was larger 0.5 cm. Leaves partially covered by a jaw were considered inside the field. For each bank, the absolute difference between the maximum position and the minimum position of all open leaves inside the field (${\text{pos}}_{\text{max}}^{\text{bank}}$) was determined such as

(B1) $${\text{pos}}_{\text{max}}^{\text{bank}}=\left|\text{max}\left({\text{pos}}^{\text{bank}}\right)\;\text{- min}\left({\text{pos}}^{\text{bank}}\right)\right|\text{.}$$

The leaf sequence variability per bank for a control point (LSV_bank_[cp]) was then calculated according to.

(B2) $${\text{LSV}}_{\text{bank}}\left[\text{cp}\right]=1‐\frac{1}{N_{F}‐1}\sum_{\text{i = 1}}^{N_{F}‐1}\frac{\left|{\text{pos}}_{i}^{\text{bank}}{\;\text{- pos}}_{\text{i + 1}}^{\text{bank}}\right|}{{\text{pos}}_{\text{max}}^{\text{bank}}},$$

where ${\text{pos}}_{i}^{\text{bank}}$ and ${\text{pos}}_{\text{i + 1}}^{\text{bank}}$ are the position of the adjacent leaf i and i + 1 inside a bank. The calculation was repeated for the bank A and the bank B. LSV[cp] for this control point was

(B3) $$\text{LSV}\left[\text{cp}\right]{\;\text{= LSV}}_{\text{bank A}}\left[\text{cp}\right]\times {\text{LSV}}_{\text{bank B}}\left[\text{cp}\right]\text{.}$$

For each control point, the open area of leaf pair was calculated as the distance between the two opposite leaves multiplied by their widths w. Only the fraction of the leaf width f inside the field was considered if a leaf pair was partially covered by a jaw. All leaf pairs not overlapping with the jaw had f = 1. The open aperture area per control point OAA[cp] was calculated according to.

(B4) $$\text{OAA}\left[\text{cp}\right]=\sum_{i=1}^{N_{p}}f_{i}w_{i}\left|{\text{pos}}_{i}^{\text{bank A}}‐{\text{pos}}_{i}^{\text{bank B}}\right|,$$

where N_p_ is the number of open leaf pair and ${\text{pos}}_{i}^{\text{bank}}$is the i^th^ position of the leaf in the bank. The aperture area variability per control point AAV[cp] was calculated as the open aperture area per control point normalized by the maximum open area over all control points for this beam,

(B5) $$\text{AAV}\left[\text{cp}\right]=\frac{\text{OAA[cp]}}{\text{max(OAA[cp])}}\text{.}$$

Since only open leaves are considered, the aperture area from this calculation differs slightly than the aperture area used for the calculation of the edge metric. Control point weights W[cp] were determined the same way as in Appendix A. The modulation complexity score for a beam MCS_beam_ was then computed according to

(B6) $${\text{MCS}}_{\text{beam}}=\sum_{\text{cp}=1}^{N}\text{AAV}\left[\text{cp}\right]\times \text{LSV}\left[\text{cp}\right]\times W\left[\text{cp}\right],$$

where N is the number of control point. Finally, MCS for the plan was determined by weighting MCS_beam_ with respect to the monitor unit MU of this beam

(B7) $$\text{MCS =}\;\sum_{\text{beam}}{\text{MCS}}_{\text{beam}}\frac{{\text{MU}}_{\text{beam}}}{{\text{MU}}_{\text{total}}}\text{.}$$

In order to understand the contribution of LSV and AAV to MCS, both quantities were evaluated independently for each plan, namely

(B8) $${\text{LSV}}_{\text{beam}}=\sum_{\text{cp}=1}^{N}\text{LSV}\left[\text{cp}\right]\times W\left[\text{cp}\right],$$

and

(B9) $${\text{AAV}}_{\text{beam}}=\sum_{\text{cp}=1}^{N}\text{AAV}\left[\text{cp}\right]\times W\left[\text{cp}\right],$$

for beams and

(B10) $$\text{LSVw =}\;\sum_{\text{beam}}{\text{LSV}}_{\text{beam}}\frac{{\text{MU}}_{\text{beam}}}{{\text{MU}}_{\text{total}}},$$

and

(B11) $$\text{AAVw =}\;\sum_{\text{beam}}{\text{AAV}}_{\text{beam}}\frac{{\text{MU}}_{\text{beam}}}{{\text{MU}}_{\text{total}}},$$

for plans, where LSVw and AAVw stand for leaf sequence variability weighted and area aperture variability weighted, respectively.

## Appendix C Sphericity

The sphericity was determined with the Pyradiomics v3.0 module. This quantity is defined as

(C1) $$\text{sphericity}=\frac{{\left(36\pi V^{2}\right)}^{1/3}}{A},$$

where V is the volume of the structure and A is the surface area of the structure. In these terms, the sphericity is a dimensionless quantity reporting value between 0 and 1, where 1 represents a perfect sphere.
